# CDPKs CPK6 and CPK3 Function in ABA Regulation of Guard Cell S-Type Anion- and Ca^2+^- Permeable Channels and Stomatal Closure

**DOI:** 10.1371/journal.pbio.0040327

**Published:** 2006-10-10

**Authors:** Izumi C Mori, Yoshiyuki Murata, Yingzhen Yang, Shintaro Munemasa, Yong-Fei Wang, Shannon Andreoli, Hervé Tiriac, Jose M Alonso, Jeffery F Harper, Joseph R Ecker, June M Kwak, Julian I Schroeder

**Affiliations:** 1 Cell and Developmental Biology Section, Division of Biological Sciences and Center for Molecular Genetics, University of California San Diego, La Jolla, California, United States of America; 2 Department of Agriculture, Okayama University, Okayama, Japan; 3 The Salk Institute of Biological Studies, La Jolla, California, United States of America; 4 Biochemistry Department, University of Nevada, Reno, Nevada, United States of America; Oregon State University, United States of America

## Abstract

Abscisic acid (ABA) signal transduction has been proposed to utilize cytosolic Ca^2+^ in guard cell ion channel regulation. However, genetic mutants in Ca^2+^ sensors that impair guard cell or plant ion channel signaling responses have not been identified, and whether Ca^2+^-independent ABA signaling mechanisms suffice for a full response remains unclear. Calcium-dependent protein kinases (CDPKs) have been proposed to contribute to central signal transduction responses in plants. However, no *Arabidopsis CDPK* gene disruption mutant phenotype has been reported to date, likely due to overlapping redundancies in CDPKs. Two *Arabidopsis* guard cell–expressed *CDPK* genes, *CPK3* and *CPK6*, showed gene disruption phenotypes. ABA and Ca^2+^ activation of slow-type anion channels and, interestingly, ABA activation of plasma membrane Ca^2+^-permeable channels were impaired in independent alleles of single and double *cpk3cpk6* mutant guard cells. Furthermore, ABA- and Ca^2+^-induced stomatal closing were partially impaired in these *cpk3cpk6* mutant alleles. However, rapid-type anion channel current activity was not affected, consistent with the partial stomatal closing response in double mutants via a proposed branched signaling network. Imposed Ca^2+^ oscillation experiments revealed that Ca^2+^-reactive stomatal closure was reduced in CDPK double mutant plants. However, long-lasting Ca^2+^-programmed stomatal closure was not impaired, providing genetic evidence for a functional separation of these two modes of Ca^2+^-induced stomatal closing. Our findings show important functions of the CPK6 and CPK3 CDPKs in guard cell ion channel regulation and provide genetic evidence for calcium sensors that transduce stomatal ABA signaling.

## Abstract 2

Two*CDPK* genes were found to be expressed in*Arabidopsis* guard cells, where they are involved in ion channel regulation in response to abscisic acid signaling.

## Introduction

Stomatal pores in the epidermis of aerial parts of plants facilitate gas exchange between plants and the atmosphere. Stomatal pores are surrounded by pairs of guard cells that mediate stomatal pore opening and closing. Guard cells respond to diverse stimuli, including blue light, CO_2_ concentrations, drought, pathogen attack, and plant hormones, including abscisic acid (ABA) [1–3]. Stomatal movements are mediated by ion transport across the plasma membrane and vacuolar membrane of guard cells and by organic solute content changes [1,4]. Guard cell ion channels and proton pumps are regulated by the cytosolic free Ca^2+^ concentration ([Ca^2+^]_cyt_) such that [Ca^2+^]_cyt_ elevation activates stomatal closing mechanisms [5–9]. These findings correlate with the Ca^2+^ dependence of ABA-induced stomatal closing [10,11].

ABA is a drought-inducible plant hormone. ABA regulates [Ca^2+^]_cyt_ elevations in guard cells [12–18] and other cells [19,20]. [Ca^2+^]_cyt_ elevation activates slow-type (S-type) anion efflux channels and down-regulates inward-rectifying K^+^ channels and proton pumps in the plasma membrane of guard cells [5,7,16,21]. The concomitant efflux of anions and K^+^ from guard cells results in turgor reduction and stomatal closure. Thus, Ca^2+^ elevation shifts ionic conductance properties in guard cells toward a stomatal closing favoring mode. Other studies indicate a possible role for Ca^2+^ in mediating stomatal opening as well [22–24]. It is noteworthy that ABA signal transduction in guard cells consists of not only a Ca^2+^-dependent response but also a Ca^2+^-independent possibly parallel pathway [25] (for reviews: [3,4], see Discussion). Whether Ca^2+^-independent mechanisms suffice for mediating full physiological stomatal ABA responses remains unclear. Genes encoding signal transduction proteins in both Ca^2+^-dependent and -independent pathways are still largely unknown and molecular genetic evidence for a rate-limiting role of Ca^2+^ as a positive transducer of ABA signaling is lacking. Repression of the *SOS3-like calcium binding protein 5* (*SCaBP5*)/*Calcineurin B-like protein 1* (*CBL1*) and *CBL9* genes result in ABA hypersensitivity, suggesting that CBL1 and CBL9 function as negative regulators of an ABA signal transduction pathway [26,27]. However, no genes encoding Ca^2+^-sensing proteins that function as positive transducers of ABA signaling and of ion channel regulation in guard cells have been identified.

ABA regulates repetitive [Ca^2+^]_cyt_ elevations in guard cells [12–15,17,28,29]. Experimentally imposing [Ca^2+^]_cyt_ transients revealed two distinguishable Ca^2+^-dependent stomatal closing responses: a rapid “Ca^2+^-reactive” stomatal closing response, and a long-lasting “Ca^2+^-programmed” stomatal closing response, which prevails even after Ca^2+^ transients have been terminated [29]. The long-lasting Ca^2+^-programmed response, but not the rapid Ca^2+^-reactive stomatal closing response, depends on the Ca^2+^ transient pattern [29–31]. However, the underlying Ca^2+^ transduction mechanisms remain unknown. In guard cells, [Ca^2+^]_cyt_ elevation activates S-type anion channels via phosphorylation events [16,32], suggesting a role for phosphorylation events in [Ca^2+^]_cyt_ signaling. We hypothesized that guard cell–expressed calcium-dependent protein kinases (CDPKs) [33] may function in transducing specific components of guard cell signal transduction and ion channel regulation.

CDPKs are protein kinases that are broadly distributed in the plant kingdom [34,35]. In other kingdoms, CDPKs have been found only in certain groups of protists, including *Plasmodium* [34]. CDPKs contain an intrinsic Ca^2+^-activation domain with four EF hand Ca^2+^-binding sites [33]. CDPKs have been proposed to function in multiple plant signal transduction pathways downstream of [Ca^2+^]_cyt_ elevations, thus transducing various physiological responses [33,34]. A virus-induced gene silencing study demonstrated a role of CDPKs in tobacco defense responses [36]. Dominant constitutively active mutant forms of *CPK10/AtCDPK1* (*Arabidopsis* gene identifier number [AGI No.] At1g74740) or *CPK30/AtCDPK1a* (AGI No. At1g18890) cause constitutive stress signaling [37], dominant mutant isoforms affect pollen tube growth in *Petunia* [38], and antisense repression of CDPK in Medicago truncatula affected root hair development and the nodule formation rate [39]. A Ca^2+^-dependent kinase activity phosphorylates the guard cell–expressing inward-rectifying K^+^ channel, KAT1, in vitro [40] and recombinant *Arabidopsis* CDPK activates Cl^−^ influx channels in Vicia faba vacuoles [41]. These results suggest functions of CDPKs in stomatal movements. Whereas many biochemical approaches show functions of CDPKs [36,37,41–50], gene disruption phenotypes of CDPKs have not yet been reported in *Arabidopsis* and would allow unequivocally pinpointing cellular functions of defined *CDPK* genes.

The CDPK gene family includes 34 members in *Arabidopsis* alone [35,46]. Redundancies in *CDPK* genes are likely to hamper molecular genetic analyses of CDPK functions. Recent studies have shown that relatively few signal transduction genes are expressed in a strictly cell-specific manner in roots, guard cells, and mesophyll cells [51–54]. However, signal transduction genes that are highly expressed in a given cell type, such as guard cells, are candidates for being incorporated into signal transduction networks within those cells. Furthermore, cell-specific signal transduction assays may allow resolution of phenotypes of single knockout mutants that global whole plant phenotypic analyses would not resolve in reverse genetic studies. Therefore, in the present study we used single cell-type gene expression analysis [54] to determine which *CDPK* genes are expressed in guard cells. We report phenotypes of loss-of-function mutants in two of the guard cell–expressed CDPKs and characterize functions of these CDPKs in Ca^2+^ activation of anion channels, in ABA activation of anion channels, and unexpectedly also in ABA regulation of Ca^2+^ channels as well as in Ca^2+^-reactive and ABA-induced stomatal closing. The presented results provide molecular genetic and cell biological evidence for Ca^2+^ sensors that function as positive transducers in plant ion channel regulation and ABA- and Ca^2+^-dependent signal transduction in guard cells.

## Results

### Expression of CDPK Genes

There are 34 *CDPK* genes in the *Arabidopsis* genome [35,46]. To investigate whether and, if so, where CDPKs may function in ion channel regulation and guard cell signal transduction branches, we first identified *CDPK* genes expressed in guard cells using a guard cell–enriched cDNA library and RT-PCR with degenerate oligomers [55]. Two of the guard cell–expressed *CDPK* genes, *CPK3* (AGI No.: At4g23650) and *CPK6* (AGI No.: At2g17290), showed initial insertion mutant phenotypes and were therefore further analyzed. The expression of *CPK3* and *CPK6* in isolated guard cell protoplasts (GCPs) was further analyzed by RT-PCR with gene-specific primers (Figure 1A) and independently later by cell type–specific genomic scale expression analyses using Affymetrix (Santa Clara, California, United States) GeneChip assays [54]. RT-PCR analysis showed that *CPK3* and *CPK6* are expressed in both guard cells and mesophyll cells (Figure 1A). The purity of GCPs was analyzed by RT-PCR with specific primers for the guard cell–expressed potassium channel gene, *KAT1* (Figure 1A) [56]. Guard cell preparations were further examined for contamination of mesophyll cells by analyzing mRNA abundance of a putative calmodulin-binding protein (*CBP*) (AGI No.: At4g33050), which was identified as being highly expressed in mesophyll cells but absent in guard cells [54]. No *CBP* mRNA was detected in guard cell preparations (Figure 1A), indicating that the GCP preparations had no or very little contamination. In addition to *CPK3* and *CPK6*, several other CDPK genes were identified in guard cells by microarray experiments with guard cell RNA (Supplemental Table I in [54]). In this study, we focus on functional dissection of the guard cell–expressed *CPK3* and *CPK6* genes.

**Figure 1:**
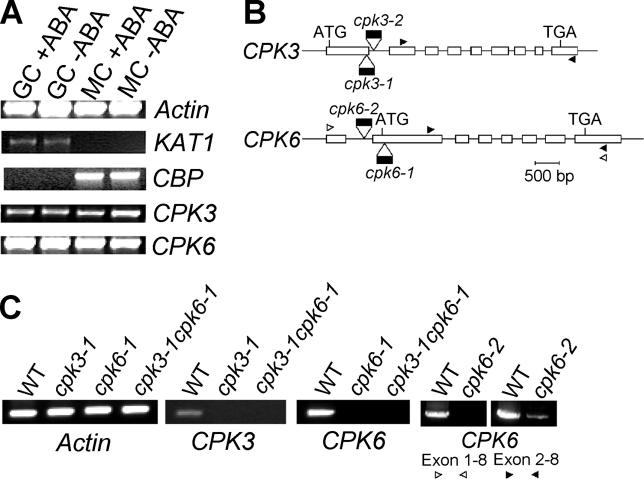
Guard Cell Expression of *CPK3* and *CPK6* CDPKs (A) Expression of *CPK3* and *CPK6* in guard cell (GC) and mesophyll cell (MC) protoplasts was examined by RT-PCR. Control amplifications of the guard cell-expressed *KAT1* gene and the mesophyll-expressed *CBP* marker genes [54] (Leonhardt et al., 2004) were used to test the purity of cell preparations (see Results). *ACTIN2* was used for an internal loading control. To amplify each *CDPK*-specific band, RT-PCR was performed with primer sets as indicated by arrowheads in (B) for 36 cycles. Plants were sprayed with water (−ABA) or 100 μM ABA (+ABA) 4 h before isolation of protoplasts and RNA extraction. (B) Cartoon showing the T-DNA insertion positions in *cpk3* and *cpk6* T-DNA insertion alleles. PCR was performed with a left boarder primer of the T-DNA and a gene-specific primer, and the PCR products were sequenced to determine the T-DNA insertion positions. Arrowheads indicate primer locations for RT-PCR in (A) and (C). ATG and TGA indicate start and stop codons. White boxes indicate exons. (C) RT-PCR confirmed that *cpk3–1* and *cpk6–1* alleles were disruption mutants. PCRs (32 cycles) were performed with primer sets as indicated in (B) (black arrowheads) in the left three panels. Transcripts of wild-type (WT) and *cpk6–2* were examined with two sets of primers [white and black arrowheads in (B)] showing that *cpk6–2* lacks exon 1 and that the *cpk6–2* has 8% or less the mRNA level of wild-type based on densitometry analyses (*n* = 2). RNA was extracted from leaves of WT, homozygous *cpk3–1*, *cpk6–1,* and *cpk6–2* single mutants, and the *cpk3-1cpk6–1* double mutant.

To genetically analyze functions of CPK3 and CPK6 in guard cell signal transduction, we identified T-DNA insertion mutations in *CPK3* and *CPK6* from the Salk Institute Genomic Analysis Laboratory database [57]. Homozygous T-DNA insertion mutant lines were isolated and genomic sequences of the *cpk3–1* (SALK_107620), *cpk3–2* (SALK_022862), *cpk6–1* (SALK_093308), and *cpk6–2* (SALK_033392) insertion mutants were determined. The T-DNA insertions in *cpk3–1* and *cpk3–2* are localized in the first exon and in the first intron, respectively (Figure 1B). The insertion in *cpk6–1* is localized in the second exon, 60 base-pairs downstream of the translation initiation codon, and *cpk6–2* is in the first intron (Figure 1B). Southern blot analyses of homozygous plants indicated only a single band in each line, suggesting a single T-DNA insertion in these mutants (data not shown). Transcripts of *CPK3* or *CPK6* were not detected in *cpk3–1* and *cpk6–1* as demonstrated by RT-PCR utilizing whole leaf RNA extracts (Figure 1C)*.* No RT-PCR band was observed for *cpk3–2* (data not shown). For *cpk6–2*, no full-length cDNA was detected (Figure 1C). A faint band for transcript downstream of the T-DNA insertion was observed after 35 cycles of amplification showing substantially reduced mRNA levels (8% or less intensity compared to wild-type level, *n* = 2) (Figure 1C). Using a primer set in the first exon and the eighth exon, no RT-PCR amplification was observed (Figure 1B and 1C), showing that the first exon is missing in *cpk6–2*. We performed RT-PCR with *CPK3* primers in *cpk6–1* and with *CPK6* primers in *cpk3–1* to examine whether a compensatory expression occurs. No compensation in the wild-type transcript levels was observed (data not shown).

Homozygous *cpk3* and *cpk6* single and *cpk3-1cpk6–1* and *cpk3-2cpk6–2* double mutants were isolated and used for further analyses. Whole plant general morphological phenotypes of *cpk3–1, cpk3–2, cpk6–1, cpk6–2,* and the *cpk3-1cpk6–1* and *cpk3-2cpk6–2* double mutants were largely similar to wild-type plants (Columbia ecotype) under the standard growth conditions tested, but *cpk3-1cpk6–1* and *cpk3-2cpk6–2* double mutant plants showed a slight delay in growth by approximately 2 d in 4-wk-old plants compared to wild-type plants (data not shown).

### Activation of S-Type Anion Channels by Cytosolic Ca^2+^ Is Impaired in *cpk3cpk6* Mutants

S-type anion efflux channels have been proposed to play an important role as targets of ABA signal transduction in guard cells and to be regulated by upstream phosphorylation events [5,9,16,32,55,58–62]. To determine whether CDPKs function in the activation of S-type anion channels in guard cells, we examined Ca^2+^ activation of S-type anion channels in wild-type, *cpk3,* and *cpk6* single mutant and double mutant guard cells.

As illustrated in Figure 2, typical large [Ca^2+^]_cyt_-activated S-type anion channel currents were observed in wild-type guard cells in the presence of elevated (2 μM) free Ca^2+^ in the patch pipette (cytosolic) solution (Figure 2A and 2C) as previously described [5,9]. At 0.1 μM [Ca^2+^]_cyt_ in the pipette, large S-type anion currents were not activated (data not shown) [9]. S-type anion channel currents in the presence of 2 μM [Ca^2+^]_cyt_ were significantly reduced in the *cpk3–1* mutant (*n* = 11 guard cells) compared to wild-type (Figure 2C; *n* = 17, *p* = 0.022 at −145 mV). *cpk3–2* showed similar results to *cpk3–1* (Figure 2C; *n* = 7, *p* > 0.4 compared to *cpk3–1*). S-type anion channel currents were further reduced in *cpk6–1* (*n* = 11) and *cpk6–2* (*n* = 4) single mutant guard cells (Figure 2C; *p* < 0.005 compared to wild-type; *p* > 0.18 for *cpk6–1* compared to *cpk6–2*). In *cpk3-1cpk6–1* double mutant guard cells (*n* = 17), S-type anion channel currents were substantially reduced compared to wild-type controls (Figure 2B and 2C; *p* < 0.0005). Essentially similar results were obtained in *cpk3-2cpk6–2* (Figure 2C; *n* = 6, *p* > 0.4 compared to *cpk3-1cpk6–1*). Nevertheless, background anion currents did remain in the double mutants (Figure 2B and 2C). The data showed stronger effects of *CPK6* disruptions compared to *CPK3* disruptions. Thus *CPK3* and *CPK6* are important for Ca^2+^ activation of S-type anion channels.

**Figure 2:**
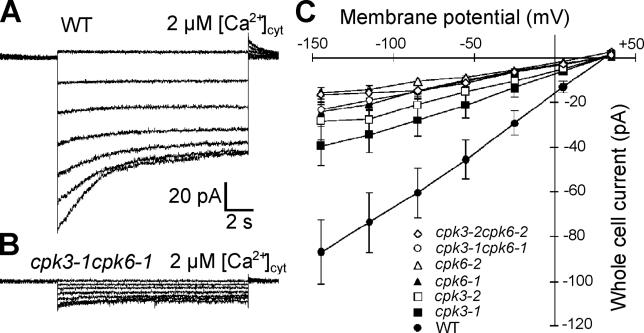
Impairment in [Ca^2+^]_cyt_-Dependent Activation of S-Type Anion Channel Currents in Guard Cells of *cpk3* and *cpk6* Single and *cpk3cpk6* Double Mutants (A and B) Typical S-type anion channel current traces in wild-type (WT) (A) and *cpk3-1cpk6–1* double mutant (B) guard cells are shown in response to 2 μM free Ca^2+^ in the patch-clamp pipette solution that dialyzes the cytoplasm. (C) Average current-voltage curves of wild-type (*n* = 17 cells), *cpk3–1* (*n* = 11), *cpk3–2* (*n* = 7), *cpk6–1* (*n* = 11), *cpk6–2* (*n* = 4), *cpk3-1cpk6–1* (*n* = 17), and *cpk3-2cpk6–2* (*n* = 11). Error bars show SEM.

### Impairment in ABA Activation of S-Type Anion Channels in *cpk3cpk6* Mutants

Previous studies have shown that guard cell signal transduction is mediated by a network of events, which includes parallel Ca^2+^-dependent and -independent signaling branches (see Discussion; for reviews: [3,4]). Therefore, experiments were pursued to analyze ABA activation of S-type anion channels. These experiments were performed under different conditions than those shown in Figure 2, such that cytosolic Ca^2+^ elevation alone would not fully activate S-type anion currents (see Materials and Methods) [9]. As shown in Figure 3, with preincubation of guard cells in low extracellular Ca^2+^, 2 μM cytosolic Ca^2+^ in the pipette activated S-type anion currents in guard cells of only intermediate amplitudes (Figure 3A). Under these conditions, ABA up-regulated S-type anion current activities in wild-type protoplasts (Figure 3A, 3B, and 3E; *n* = 8). In *cpk3-1cpk6–1* (*n* = 7) and *cpk3-2cpk6–2* (*n* = 7) double mutant guard cells, ABA regulation of S-type anion currents was impaired (Figure 3C, 3D, and 3E; *p* < 0.01). These data show that, despite the complex network of ion channel regulation mechanisms in guard cells [3], CDPKs mediate an important Ca^2+^-decoding transduction step in ABA regulation of S-type anion channels (Figures 2 and 3). ABA activation in wild-type guard cells and the impairment in ABA activation of S-type anion channels at 2 μM cytosolic Ca^2+^ in *cpk3cpk6* mutants (Figure 3) further provide evidence for a recently proposed hypothesis in which stomatal closing signals (i.e., ABA) mediate priming of guard cell Ca^2+^ sensors, such that they can respond to elevated cytosolic Ca^2+^ levels [63].

**Figure 3:**
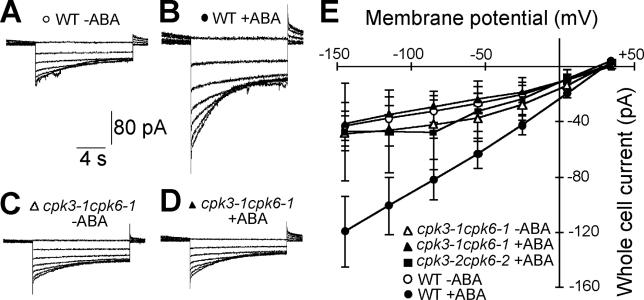
Impairment in ABA Activation of S-Type Anion Channel Currents in *cpk3cpk6* Mutant Guard Cells (A and B) Whole-cell S-type anion channel current traces in wild-type (WT) guard cells in the absence of ABA (A) and in the presence of 50 μM ABA (B). (C and D) Whole-cell S-type anion channel current traces in *cpk3-1cpk6–1* double mutant guard cells in the absence of ABA (C) and in the presence of ABA (D). (E) Average current-voltage curves of wild-type and *cpk3-1cpk6–1* and *cpk3-2cpk6–2* double mutant guard cells in the absence and presence of ABA (*n* = 8 WT; *n* = 7 *cpk3-1cpk6–1*; *n* = 3 *cpk3-2cpk6–2* guard cells). GCPs were treated with 50 μM ABA or solvent control (0.1% ethanol) for 2 h prior to establishing gΩ seals. Open and closed symbols indicate −ABA and +ABA, respectively.

### Impairment in ABA activation of I_Ca_ Channels in *cpk3 cpk6* Mutants

ABA activates plasma membrane Ca^2+^-permeable (I_Ca_) channels [64–66]. Combined physiological, molecular genetic, and cell biological analyses have shown that I_Ca_ channels function in the guard cell ABA signal transduction network at hyperpolarized voltages [9,53,64–66]. We examined whether *CPK3* and *CPK6* function in the regulation of I_Ca_ channels.

Typical I_Ca_ currents were activated by extracellular application of ABA to patch-clamped wild-type guard cells (Figure 4A and 4B, *n* = 13). Unexpectedly, ABA activation of I_Ca_ channels was not observed in *cpk3-1cpk6–1* and *cpk3-2cpk6–2* double mutant guard cells (Figure 4C–4E; *n* = 14, *p* = 0.56 for *cpk3-1cpk6–1*; *n* = 8, *p* = 0.47 for *cpk3-2cpk6–2* when comparing before and after ABA treatment). Blind patch-clamp experiments in which the genotype of protoplasts was unknown (*n* = 2 for wild-type, *n* = 2 for *cpk3-1cpk6–1*), and similar findings by Y.M., I.C.M., Y.W., and S.M. in this study, further confirmed the impairment of ABA activation of I_Ca_ channels in *cpk3cpk6* mutant guard cells. Next we analyzed whether only one of the two CDPKs might affect ABA activation of I_Ca_ channels. Defects in the ABA activation of I_Ca_ channels were observed in the *cpk3–1*, *cpk3–2*, *cpk6–1,* and *cpk6–2* single mutants (Figure 4F–4I; *n* = 9, *p* = 0.38 for *cpk3–1*; *n* = 11, *p* = 0.47 for *cpk6–1*; *n* = 3, *p* = 0.96 for *cpk3–2*; *n* = 5, *p* = 0.30 for *cpk6–2,* when comparing before and after ABA-treatment). Together these data show that *CPK3* and *CPK6* function in ABA regulation of I_Ca_ channels (Figure 4) and Ca^2+^ and ABA activation of S-type anion channels (Figures 2 and 3).

**Figure 4:**
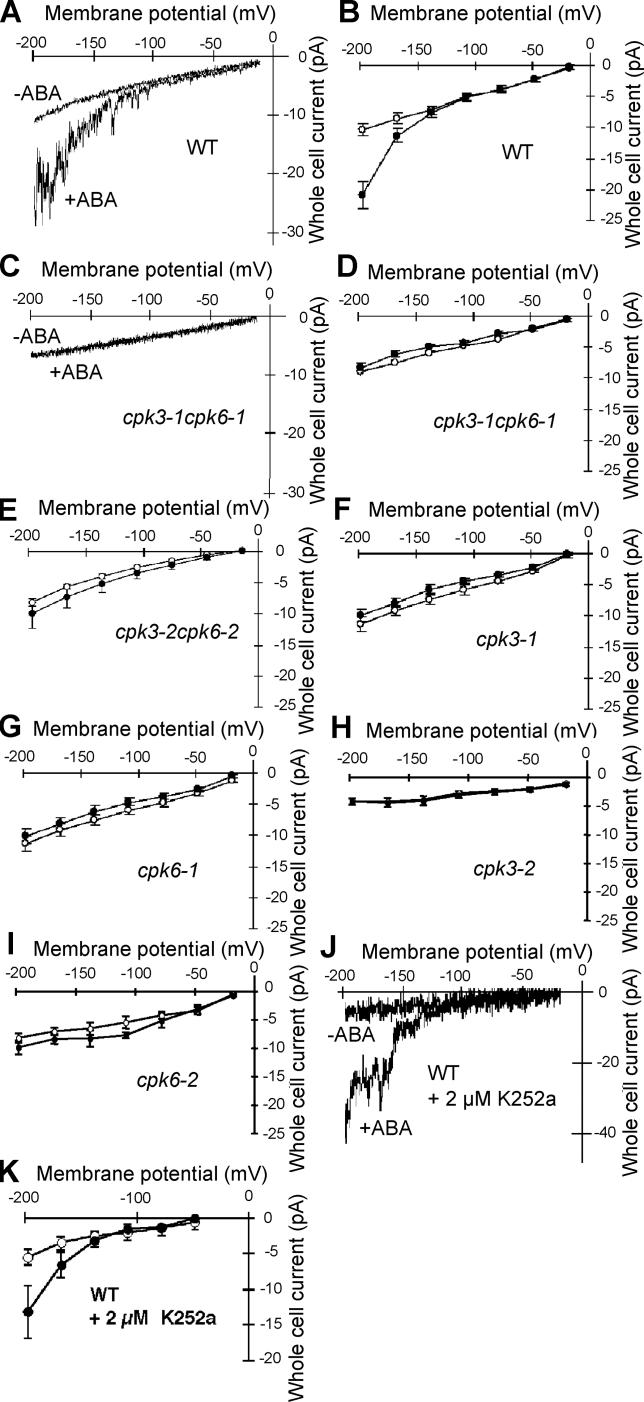
Impairment in ABA Activation of I_Ca_ Channel Currents in *cpk3* and *cpk6* Single Mutants and *cpk3cpk6* Double Mutants (A and B) ABA (50 μM) activated I_Ca_ channel current in wild-type (WT) guard cells. (A) Current traces before (−ABA) and after ABA (+ABA) activation of I_Ca_ channels are shown in a representative cell. (B) Average current-voltage curves (*n* = 13) are shown. (C and D) ABA failed to activate I_Ca_ channel currents in *cpk3-1cpk6–1* double mutant guard cells. (C) A response in a representative cell is shown. (D) Average current-voltage curves (*n* = 14) are shown. Traces before and after ABA overlap in (C). (E–I) Averages of current-voltage curves in guard cells isolated from (E) *cpk3-2cpk6–2* double mutant (*n* = 8), (F) *cpk3–1* (*n* = 11), (G) *cpk6–1* (*n* = 9), (H) *cpk3–2* (*n* = 3), and (I) *cpk6–2* (*n* = 5) are shown. (J and K) ABA activation of I_Ca_ channel currents in wild-type (WT) guard cells pretreated with the protein kinase inhibitor K252a (2 μM) for 15 min prior to and during patch clamping and with no ATP added in the patch pipette solution. Response in a guard cell is shown in (J), and average current-voltage curves (*n* = 6) are shown in (K). Open symbols indicate −ABA and closed symbols indicate +ABA. Error bars represent SEM.

To gain insight into the question whether activation of phosphorylation events are required before or after ABA application and during ABA activation of I_Ca_ channels in patch-clamped *Arabidopsis* guard cells, wild-type guard cells were pretreated with the broad serine/threonine kinase inhibitor, K252a [(8R*,9S*,11S*)-(−)-9-hydroxy-9-methoxycarbonyl-8-methyl-2,3,9,10-tetrahydro-8,11,epoxy-1*H*,8*H*,11*H*-2,7*b*,11*a*-triazadibenzo[*a,g*]cyclo-octa[*c,d,e*]-trinden-1-one], 20 min prior to, and continuously during, patch clamping. Moreover, the cytoplasm of whole cells was dialyzed in the absence of ATP in whole cell recordings for longer than 15 min prior to extracellular ABA exposure. Ionic currents were recorded in the same guard cells prior to and after exposure to ABA. In negative controls, treatment with K252a alone did not cause constitutive activation of I_Ca_ channel currents without ABA treatment (*n* = 3). Interestingly, pretreatment with K252a (2 μM) did not disrupt ABA activation of I_Ca_ channels (Figure 4J and 4K; *n* = 6, *p* < 0.04 at −180 mV). These findings indicate that the effect of the *cpk3* and *cpk6* mutations on ABA activation of I_Ca_ channel currents are most likely not caused by direct ABA-induced upstream CDPK activation in the NADPH-dependent activation branch of I_Ca_ channels [66], but possibly by prior CDPK action (see Discussion).

We further examined whether the defect in ABA activation of I_Ca_ channels is due to impairment in reactive oxygen species (ROS) activation of I_Ca_ channels, as found for the ABA-insensitive mutants, *gca2* and *abi2–1* [65,66]. As shown in Figure 5, hydrogen peroxide activation of I_Ca_ channels was observed in *cpk3cpk6* double mutant and wild-type guard cells (Figure 5; *n* = 7, *p* < 0.001 for *cpk3-1cpk6–1*; *n* = 4, *p* < 0.05 for *cpk3-2cpk6–2;* and *n* = 7, *p* < 0.01 for wild-type; when comparing before and after H_2_O_2_ treatment).

**Figure 5:**
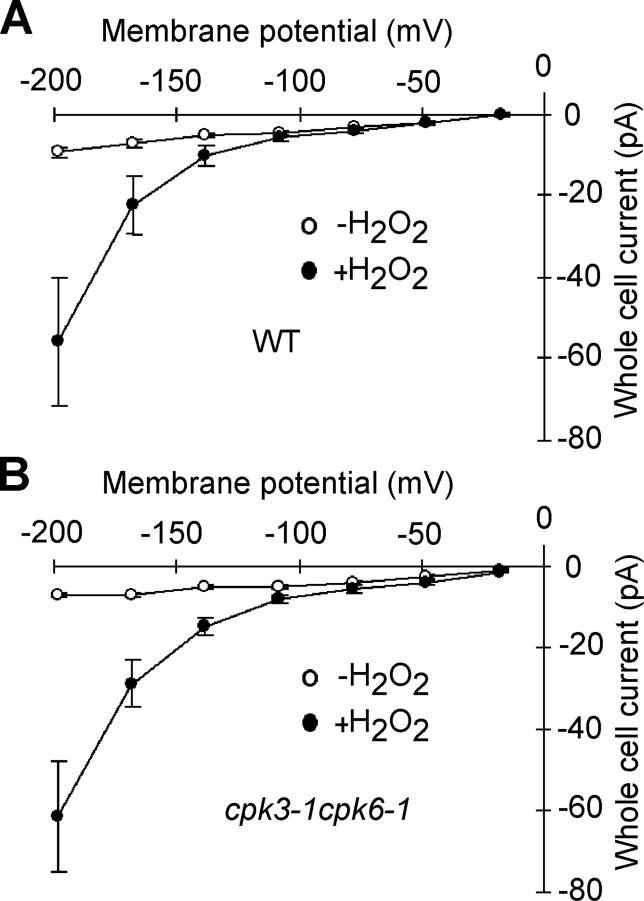
Hydrogen Peroxide Activates I_Ca_ Channels Both in Wild-type and *cpk3cpk6* Double Mutant Guard Cells (A and B) Exogenous H_2_O_2_ (5 mM) activated I_Ca_ channels in (A) wild-type (WT) (*n* = 9) and (B) *cpk3-1cpk6–1* double mutant guard cells (*n* = 12). Error bars represent SEM.

### ABA- and Ca^2+^-Induced Stomatal Closure

The findings that cytosolic Ca^2+^ and ABA activation of S-type anion channels and ABA activation of I_Ca_ channels are impaired in *cpk3cpk6* mutants led us to examine ABA-induced stomatal closure in these *cdpk* mutants. Application of 1 and 10 μM ABA induced a decrease in stomatal aperture in wild-type (Figure 6A, *n* = 13 experiments, 260 stomata). In contrast, *cpk3-1cpk6–1* and *cpk3-2cpk6–2* double mutant stomata showed reduced ABA responses (Figure 6A, Table 1, *n* = 7 experiments, 140 stomata; at 10 μM ABA: *p* < 0.01 for *cpk3-1cpk6–1* versus wild-type; *n* = 4 experiments, 120 stomata, *p* < 0.011 for *cpk3-2cpk6–2*). Furthermore, ABA-induced stomatal closing was partially impaired in all of the *cpk3* and *cpk6* single mutant alleles (*p* < 0.05) (Table 1). These results show roles for *CPK3* and *CPK6* in ABA-induced stomatal closure.

**Figure 6:**
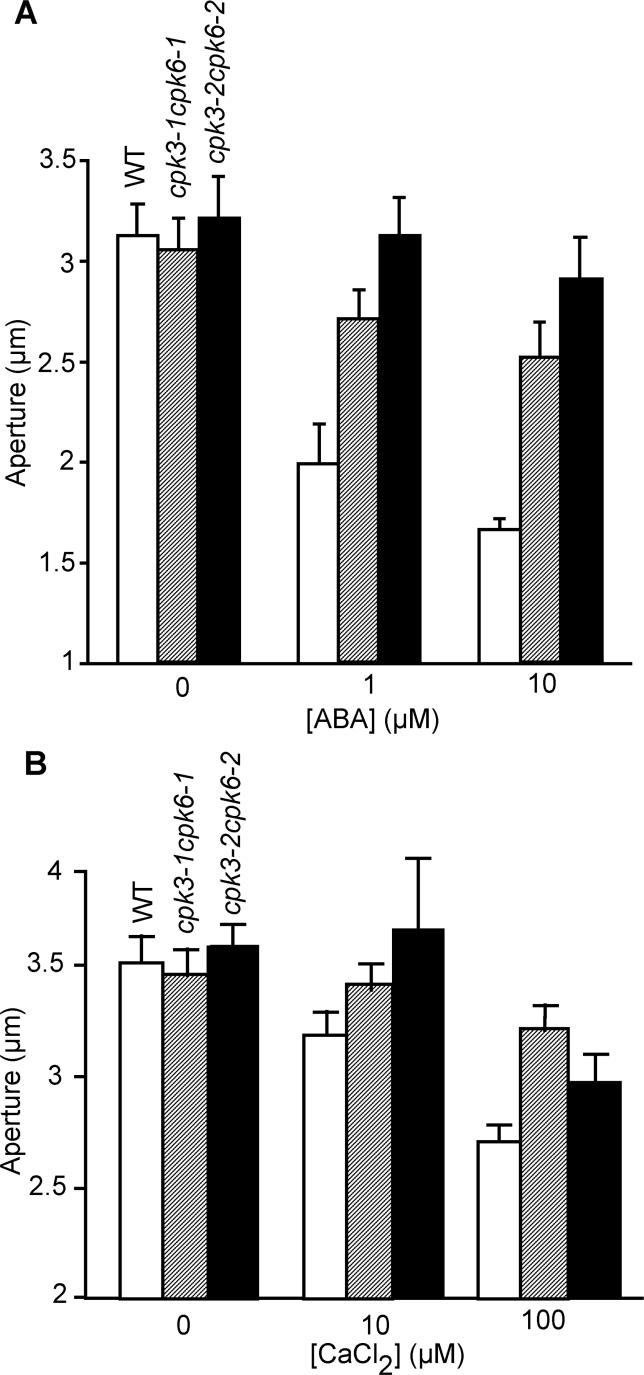
ABA- and Ca^2+^-Induced Stomatal Closure Is Partially Impaired in *cpk3cpk6* Mutants (A) ABA-induced stomatal closing (white bars: wild-type; shaded bars: *cpk3-1cpk6–1* double mutant; black bars: *cpk3-2cpk6–2* double mutant). Average data from representative experiments are shown (wild-type: WT, *n* = 13 experiments, 260 total stomata; *cpk3-1cpk6–1* double mutant: *n* = 7 experiments, 140 stomata; *cpk3-2cpk6–2 n* = 4 experiments, *n* = 120 stomata). (B) External Ca^2+^-induced stomatal closing (white bars: wild-type, shaded bars: *cpk3-1cpk6–1* double mutant, black bars: *cpk3-2cpk6–2* double mutant). Average data from representative experiments are shown (wild-type: WT, *n* = 9 experiments including 4 blind experiments, 180 total stomata; *cpk3-1cpk6–1* double mutant, *n* = 9 experiments including 4 blind experiments, 180 total stomata; *cpk3-2cpk6–2*, *n* = 4 experiments, *n* = 80 stomata). Stomatal aperture widths are illustrated. Error bars represent SEM.

**Table 1:**
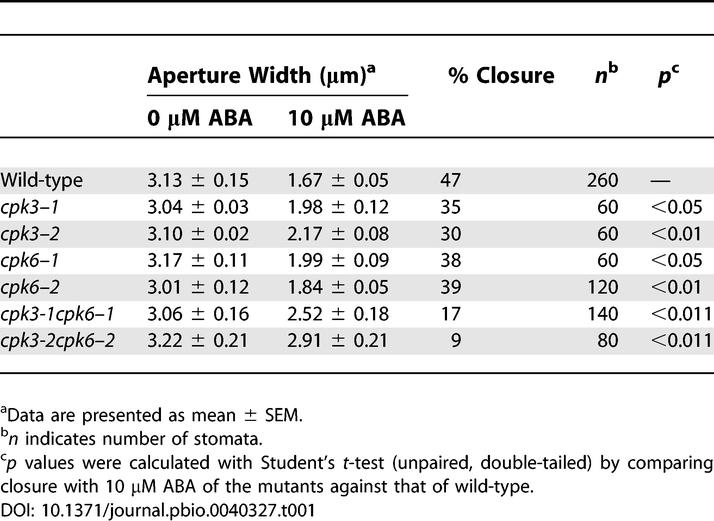
ABA-Induced Stomatal Closure in *cpk3* and *cpk6* Mutants

Extracellular Ca^2+^ causes stomatal closing, by initiating repetitive cytoplasmic Ca^2+^ elevations in guard cells [67–69]. Application of 100 μM CaCl_2_ to intact leaves closed wild-type stomata (Figure 6B). However, in the *cpk3-1cpk6–1* and *cpk3-2cpk6–2* double mutants, stomatal closure was significantly attenuated compared to the wild-type response (Figure 6B, *p* < 0.01 for *cpk3-1cpk6–1* and *p* < 0.05 for *cpk3-2cpk6–2* at 100 μM external Ca^2+^). External Ca^2+^-induced stomatal closing was also impaired in all four *cpk3* and *cpk6* single mutants (*n* = 60 to 100 stomata; *p* < 0.05, data not shown).

To further evaluate Ca^2+^ regulation of stomatal closure, we examined the effect of experimentally imposed [Ca^2+^]_cyt_ oscillations on stomatal closure in *cpk3cpk6* double mutant plants [29–31,68]. A Ca^2+^ oscillation pattern was applied to guard cells with a similar time pattern to those that cause *long-term programmed* stomatal closure (i.e., inhibition of stomatal reopening) in *Arabidopsis* [29]. Hypothesizing a contribution of additional CDPKs or other Ca^2+^ transducers in these experiments, we applied a hyperpolarizing buffer with lower extracellular Ca^2+^ concentrations (1 mM) and higher KCl concentrations (1 mM) than those used in previous research [29] with the goal of experimentally imposing weaker cytosolic Ca^2+^ transients in guard cells (see Materials and Methods). Ca^2+^ imaging experiments of guard cells showed that this protocol causes cytosolic Ca^2+^ oscillations in *cpk3-1cpk6–1* double mutant and in wild-type guard cells (Figure 7, inset). The average amplitudes of imposed [Ca^2+^]_cyt_ transients were similar in wild-type and *cpk3-1cpk6–1* double mutant guard cells (*p* = 0.25; wild-type: 0.725 ratio units [RU] ± 0.043 SEM, *n* = 16 wild-type cells; *cpk3-1cpk6–1*: 0.766 RU ± 0.04 SEM, *n* = 24 *cpk3-1cpk6–1* guard cells). The average integrated total [Ca^2+^]_cyt_ ratio increase per period was also determined and found to be similar for wild-type and *cpk3-1cpk6–2* guard cells (*p* = 0.46; wild-type: 8.535 RU * 0.1 min ± 0.497, *n* = 16 wild-type cells; *cpk3-1cpk6–1*: 8.456 RU * 0.1 min ± 0.517, *n* = 24 *cpk3-1cpk6–1* cells). These data are consistent with findings that external Ca^2+^-induced [Ca^2+^]_cyt_ elevations in guard cells include intracellular Ca^2+^ release from guard cell organelles [67,69].

**Figure 7:**
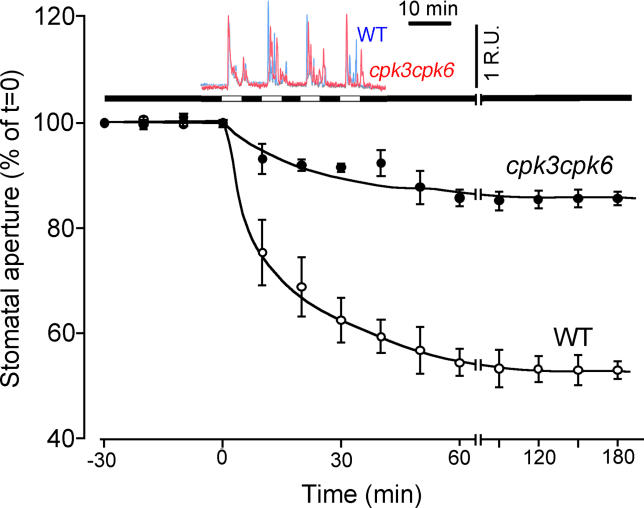
Ca^2+^-Reactive Closure Is Impaired in *cpk3cpk6* Double Mutant Stomata Four [Ca^2+^]_cyt_ transients (inset) were imposed in wild-type (WT) (*n* = 6 experiments) and *cpk3-1cpk6–1* double mutant (*n* = 6 experiments) stomata. Individually mapped stomatal apertures were measured for the last 30 min before imposed [Ca^2+^]_cyt_ transients (before Time = 0) and for the ensuing 180 min at the indicated time points. Error bars represent SEM. Inset (top) shows imposed [Ca^2+^]_cyt_ transients in wild-type (blue trace) and in *cpk3-1cpk6–1* (red trace) guard cells expressing Yellow Cameleon 3.6 (R.U.: ratio units).

Four Ca^2+^ transients with a 10-min period, which induce long-lasting “Ca-programmed” stomatal closure [29], were applied to wild-type and *cpk3cpk6* double mutant stomata. Wild-type stomata started closing immediately after the first Ca^2+^ elevation was imposed and continued to show progressive Ca^2+^-reactive closing for longer than 40 min (Figure 7; *n* = 6 experiments, 47% closure). In contrast, Ca^2+^ transient-induced closure of *cpk3-1cpk6–1* and *cpk3-2cpk6–2* double mutant stomata was reduced (14% and 22 % closure, respectively) (Figure 7; *n* = 6 experiments, *p* < 0.01 for *cpk3-1cpk6–1* versus wild-type and *n* = 3 experiments, *p* < 0.05 for *cpk3-2cpk6–2* versus wild-type, *p* > 0.60 *cpk3-1cpk6–1* versus *cpk3-2cpk6–2*). Stomata of both wild-type and *cpk3cpk6* double mutant guard cells remained closed during the ensuing 2 h and 20 min measurements, even though cells were extracellularly bathed in a typical “stomatal opening” solution containing 50 mM KCl and 0 mM CaCl_2_ and exposed to white light (Figure 7, from 40 to 180 min). Interestingly, the partial stomatal closure of *cpk3cpk6* double mutants was also maintained during the ensuing 2 h and 20 min after Ca^2+^ transients were terminated, i.e. a significant stomatal closure was observed at 180 min when compared with 0 min in both *cpk3-1cpk6–1* and *cpk3-2cpk6–2* (*p* < 0.01 for both mutants). Thus, the rapid Ca^2+^-reactive stomatal closure response is clearly impaired in the *cpk3cpk6* double mutants, whereas the long-term Ca^2+^-programmed stomatal closure response [29–31] appears to be functional (Figure 7).

Seed germination analyses were also pursued with wild-type and the *cpk3-1cpk6–1* and *cpk3-2cpk6–2* double mutants. No significant difference in ABA inhibition of seed germination in wild-type and the *cpk3cpk6* double mutant alleles was observed in the presence of 0, 0.3, 1, and 5 μM ABA after 3, 5, 7, and 11 d (unpublished data).

### Rapid-Type Anion Channel Activity Is Not Greatly Altered in *cpk3cpk6* Double Mutant

ABA also regulates a second class or mode of anion channels in guard cells, the rapid-type (R-type) anion channels [62,70–73]. Therefore, we also compared R-type anion channel current properties in wild-type and *cpk3cpk6* guard cells (Figure 8). The selectivity for anions over cations of these ion currents (Figure 8A) in *Arabidopsis* guard cells was further analyzed. Replacing Ba^2+^ with the impermeable cation tetraethylamonium in the bath solution did not affect these R-type inward currents, as previously shown [65]. Moreover, use of the impermeable anion gluconate in the pipette solution abolished the current (*n* = 3, data not shown), confirming that the recorded currents are R-type anion currents. Interestingly, in contrast to S-type anion channels (Figures 2 and 3), no significant difference was observed in the rapid anion channel activity between wild-type and *cpk3-1cpk6–1* double mutant guard cells (Figure 8A and 8B; wild-type, *n* = 7; *cpk3-1cpk6–1*, *n* = 7; *p* = 0.21 at peak current). Thus, *cpk3cpk6* mutant guard cells did not significantly impair R-type anion currents, which correlated with the partial ABA-induced stomatal closing in *cpk3cpk6* mutant guard cells (Figure 6, Table 1).

**Figure 8:**
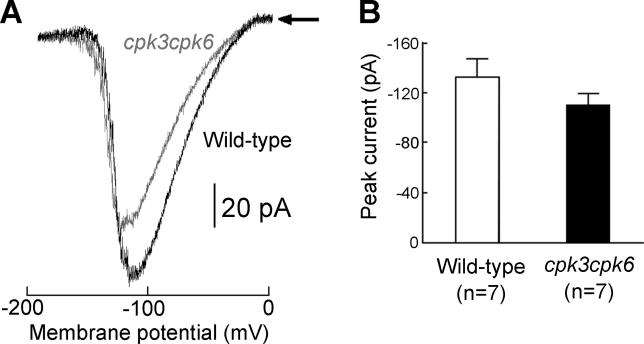
R-Type Anion Channel Currents in *cpk3cpk6* Double Mutant and Wild-Type Guard Cells (A) Representative traces of R-type anion channel current in wild-type (black trace) and *cpk3-1cpk6–1* double mutant (gray trace). (B) Averages of peak currents of R-type anion channel currents in wild-type (white bar, *n* = 7) and *cpk3-1cpk6–1* double mutant (black bar, *n* = 7 ± SEM) guard cells.

## Discussion

CDPKs have been predicted to function in response to cytoplasmic Ca^2+^ elevations in many physiological processes in plants [33,34]. Many biochemical studies have suggested roles of CDPKs in plant biology [36,40,41,43,45,48–50,74]. A dominant mutant study [37] and a biochemical study of CPK32 [49] have provided evidence for functions of CDPKs in ABA signaling, and gene-silencing/antisense *cdpk* mutant phenotypes were demonstrated for a plant defense response in tobacco [36] and for nodule formation in Medicago truncatula root hairs [39]. The relative dearth of genetic or reverse genetic *CDPK* loss-of-function mutation phenotypes may be attributed to partial redundancies in the functions of the large *CDPK* gene family. Furthermore, cell-specific and mechanistic protein regulation (e.g., ion channel) analyses allow resolution of quantitative single *cpk* gene disruption mutant phenotypes, as demonstrated here.

Our findings do not exclude the possibility that the CDPKs analyzed here may function in other pathways in other plant tissues. Single cell-type microarray studies have shown that relatively few genes are expressed in a strictly cell-specific manner in *Arabidopsis* root cell types, guard cells, and mesophyll cells [52,54]. Combinatorial usage of a single protein in different signaling pathways was initially documented in yeast [75]. Furthermore, studies of mitogen-activated protein kinase and protein phosphatase genes in plants have suggested that individual mitogen-activated protein kinases and protein phosphatases function in different signal transduction cascades in different tissues and under different conditions [55,76–79] (for review: [80]), and therefore the present study does not exclude that CPK3 and CPK6 have additional functions in other tissues. The present study demonstrates important functions of the CPK3 and CPK6 CDPKs in control of stomatal movements and in ABA and Ca^2+^ regulation of guard cell S-type anion channels and unexpectedly also in ABA regulation of Ca^2+^-permeable I_Ca_ channels.

Although many studies have shown Ca^2+^-dependent steps in guard cell signal transduction and in plant ion channel regulation [81], Ca^2+^ sensor-encoding genes that function as positive transducers in these responses have remained unknown. A calcineurin-like Ca^2+^ sensor, *CBL1/SCaBP5,* has been reported to function as a negatively regulating Ca^2+^ sensor in ABA signaling in guard cells ([26], but see [82]). Knock-out mutation of another *CBL*, *CBL9*, resulted in ABA hypersensitive phenotypes in seed germination, root and shoot growth, and ABA-inducible gene expression in *Arabidopsis*, indicating its function as a negative regulator of ABA signaling [27]. In the present study, guard cell expression analyses of *CDPK* genes aided in narrowing the number of candidate genes that may function in this system. Furthermore, the guard cell system has also allowed us to pursue detailed mechanistic and cellular analyses revealing functions of specific CDPKs in planta.

### CDPKs Function in Ca^2+^-Reactive Stomatal Closure and S-Type Anion Channel Activation

[Ca^2+^]_cyt_ elevation in guard cells causes a rapid “Ca^2+^-reactive” stomatal closure response, irrespective of the above threshold Ca^2+^ elevation pattern [29]. In contrast, a long-term “Ca^2+^-programmed” stomatal closure is modulated by imposed Ca^2+^ transient parameters [29–31]. Ca^2+^-dependent stomatal movements were analyzed in response to imposed Ca^2+^ transients. In *cpk3cpk6* double mutant plants, Ca^2+^-reactive stomatal closure was significantly reduced compared to wild-type (Figure 7). However, the partial Ca^2+^ transient-induced stomatal closing response of *cpk3cpk6* double mutant plants was maintained in white light more than 2 h after Ca^2+^ transients were terminated. Thus, the imposed long-term Ca^2+^-programmed stomatal closure response [29] appears to be functional in the *cpk3cpk6* double mutant, whereas the rapid Ca^2+^-reactive stomatal closure response is clearly impaired (Figure 7). The presented data provide genetic evidence for a mechanistic separation of Ca^2+^-reactive and Ca^2+^-programmed stomatal closure. Several other *CDPK* transcripts are expressed in guard cells as determined by oligonucleotide-based microarray experiments [54]. It is possible that other guard cell–expressed CDPKs function in Ca^2+^-programmed stomatal closure.

Previous studies have provided evidence that phosphorylation events function in Ca^2+^ activation of S-type anion channels [16,32] (see Introduction). The cytosolic Ca^2+^ activation of S-type anion channels requires the presence of hydrolyzable ATP in the patch-clamp electrode, and this channel activation is abolished by the general serine/threonine protein kinase inhibitors K252a and staurosporine [16]. The finding that Ca^2+^ activation of S-type anion channels is impaired in *cpk3* and *cpk6* gene disruption mutants provides molecular genetic evidence for this model and suggests that CDPKs function in [Ca^2+^]_cyt_ perception upstream of S-type anion channel activation (Figure 9). The *cpk6–1* and *cpk6–2* alleles exhibit similar defects in all of the phenotypes that were examined, suggesting that both alleles are strong alleles. Although both alleles show no expression of full-length cDNA, a faint band representing residual transcript of the *CPK6* open reading frame was amplified in the *cpk6–2* allele (8% or less of wild-type level). However, the *cpk6–2* transcript is lacking the wild-type origin of transcription. Furthermore, this transcript also lacks the first exon of the wild-type cDNA, and 5′ UTRs have been shown to play roles in enhancing translational efficiency [83]. Together, these data indicate that the greatly reduced level of *cpk6–2* transcripts and the absence of the first exon are sufficient to cause the strong phenotypes observed in this study. Future direct investigation of the model that CDPKs phosphorylate important targets during S-type anion channel activation will require identification of the molecular components that encode these ion channels or upstream regulators. CPK3 and CPK6 may have distinct functions in the upstream ion channel regulation pathways. The present findings point to a model in which Ca^2+^ activation of S-type anion channels functions in the rapid Ca^2+^-reactive stomatal closure response (Figures 2, 7, and 9) [29].

**Figure 9:**
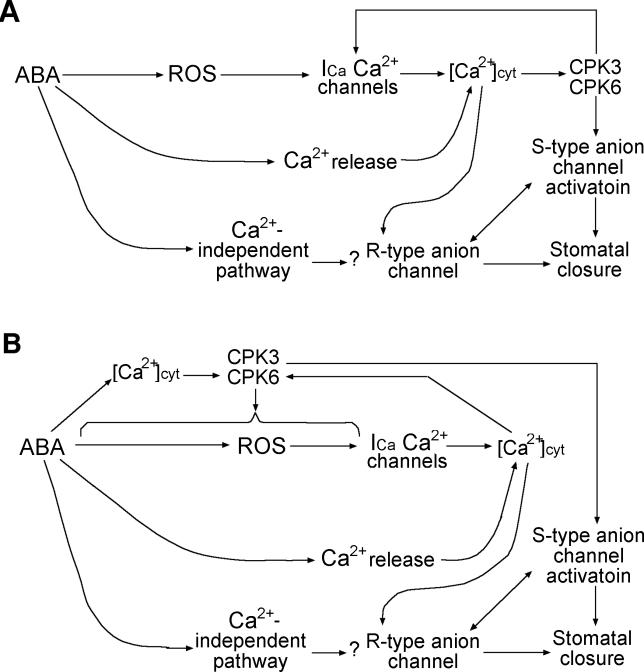
Simplified Models for Early ABA Signal Transduction Network and *CPK3* and *CPK6* Functions in Guard Cells Plasma membrane I_Ca_ channel and intracellular Ca^2+^-release mechanisms cause cytosolic Ca^2+^ elevations. *CPK3* and *CPK6* mediate [Ca^2+^]_cyt_ activation of S-type anion channels. *CPK3* and *CPK6* regulate I_Ca_ Ca^2+^ channels by a proposed feedback (A) or parallel pathway (B) or by transcriptional/translational regulation mechanism (see Discussion). R-type anion channels are regulated in a parallel *CPK3-CPK6*-independent signal transduction pathway. The arrow connecting R-type and S-type anion channels indicates that these channels may share molecular components [89]. See Discussion for details.

### CDPKs, R-Type Anion Channels, and Ca^2+^-Independent Signaling

A Ca^2+^-independent ABA signal transduction branch has been implicated in guard cells, based on data in which ABA-induced cytosolic Ca^2+^ increases were not observed in guard cells [13,16,25,28,84–86] (for reviews: [2–4,87]). Ca^2+^-dependent or -independent ABA signaling pathways have been reported to be emphasized depending on physiological conditions in Commelina communis [25]. The abolishment of ABA-induced stomatal closing by injection of the Ca^2+^ chelator BAPTA into guard cells [86,88] indicates that a Ca^2+^-independent branch within the ABA signaling network would interact with Ca^2+^-dependent mechanisms. The present study provides direct molecular genetic evidence that Ca^2+^ sensors function within the *Arabidopsis* guard cell ABA signaling network (Figures 3, 4, and 6A) and provides a genetic basis to analyze interactions with a Ca^2+^-independent pathway.

Several studies have indicated a role for R-type anion channels in stomatal closure [62,70–73,89], in addition to S-type anion channels. ABA activation of R-type anion channels was recently directly demonstrated in a subset of Vicia faba guard cells [62,73]. Direct cytosolic Ca^2+^ activation of R-type channels has not yet been shown, although R-type anion channels in V. faba guard cells have been proposed to be Ca^2+^ activated based on external CaCl_2_ activation [71]. In *cpk3cpk6* double mutant guard cells, R-type anion channel activity was not greatly impaired under the imposed conditions (Figure 8). The partial closure of stomata in response to ABA (Figure 6A, Table 1) may thus be explained by overlapping physiological functions of R-type and S-type anion channels (Figure 9). In the present study and in a recent study, R-type anion currents were recorded in patch-clamped *Arabidopsis* (Figure 8) and V. faba [86] guard cells at resting [Ca^2+^]_cyt_ and were proposed to be activated in a Ca^2+^-independent manner in V. faba. However, in both studies, guard cells were preincubated in a solution containing 40 mM CaCl_2_ or more prior to patch clamping which would elevate Ca^2+^ in guard cells [16,67,69], and therefore Ca^2+^-independent R-type anion channel activation (Figure 9) will require further investigation. The presented *cpk3cpk6* mutant data provide first genetic evidence that parallel pathways function in regulation of R-type and S-type anion channels (Figures 2, 3, and 8), even though these anion channels may share molecular components [89] (Figure 9).

Residual small S-type anion channel currents were consistently observed in *cpk3cpk6* double mutant guard cells (Figures 2B, 2C, 3C and 3D), which should also contribute to partial ABA- and Ca^2+^-induced stomatal closing. The rate of anion efflux from *Arabidopsis* guard cells can be estimated, to predict whether the residual anion currents in *cpk3cpk6* double mutants can close stomata within a physiological response. Cell volume of *Arabidopsis* GCPs was estimated at 0.11 pL according to the average diameter of protoplasts (6 μm). An estimated Cl^−^ concentration decrease from 400 mM to 100 mM during ABA-induced stomatal closure would correspond to Cl^−^ efflux of 33.9 fmol/cell. The membrane potential of guard cells treated with ABA was approximately −50 mV [73]. Cl^−^ efflux currents in wild-type and *cpk3cpk6* double mutant guard cells at −50 mV were approximately −60 pA and −25 pA, respectively (Figure 3E). These S-type anion currents correspond to Cl^−^ efflux rates of 37.3 fmol/min for wild-type and 15.54 fmol/min for the double mutant. This estimate shows that during the slow onset of drought that occurs over several days in intact plants, the residual S-type and R-type anion currents in *cpk3 cpk6* double mutants should be sufficient for a physiological stomatal closing response. Similarly, previous estimates have predicted that ion channel activities are over 10-fold higher in biological membranes than required for a typical response, due to their high transport rate [90]. This estimate also highlights the power of analyzing individual ion channel targets for characterizing CDPK functions in plants.

It is possible that additional CDPKs contribute to activation of the residual S-type anion current activity. The partial Ca^2+^-induced stomatal closure in the *cpk* mutants (Figures 6B and 7) can be explained by Ca^2+^ activation of residual S-type anion channel currents. In summary, the lack of R-type anion channel activity alteration and the residual S-type anion current in *cpk3cpk6* mutant guard cells (Figures 2, 3, and 8) correlate with the partial ABA- and Ca^2+^-induced stomatal closing responses found here (Figures 6, 7, and 9) and may also reflect a contribution of parallel Ca^2+^-independent and/or pH-dependent [91,92] signaling mechanisms.

### CDPK Mutants Impair ABA Activation of Ca^2+^-Permeable Channels

Unexpectedly, ABA activation of I_Ca_ channel currents was impaired in *cpk3cpk6* double mutant guard cells (Figure 4). Impairment in ROS signaling partially impairs rather than abolishes ABA-induced stomatal movement responses, consistent with their activity mainly at hyperpolarized voltages [53]. The present findings are consistent with a model in which additional signaling branches function in the ABA signal transduction network parallel to the ABA → ROS → I_Ca_ signaling branch (Figure 9) [65]. Several parallel pathways function in the ABA signaling network, including intracellular Ca^2+^-release mechanisms (Figure 9) [15,17,18,81].

Interestingly, in wild-type guard cells, ABA could activate I_Ca_ channels even when cells were preexposed to the general serine/threonine kinase inhibitor K252a and the cytosol of guard cells was dialyzed with an ATP-free solution for longer than 15 min in whole-cell recordings prior to ABA exposure (Figure 4J and 4K). K252a inhibits CDPK activities in diverse plants [45,93–95] and abolishes Ca^2+^ activation of S-type anion channels in guard cells [16,32]. These data indicate the following possible mechanisms by which the *cpk3cpk6* mutant alleles may impair ABA activation of I_Ca_ channels in guard cells (Figure 4):

1. CPK3 and CPK6 may not be directly activated in response to exogenous ABA within the ABA → NADPH oxidase → ROS → I_Ca_ channel signaling branch (Figure 9). But these CDPKs are necessary for maintaining I_Ca_ channel activation in a parallel regulation pathway of I_Ca_ channels [96] that also regulates S-type anion channels (Figure 9B). In this model, ABA regulation of S-type anion channels is not strictly downstream of I_Ca_ channels (Figure 9B).

2. CDPKs may regulate I_Ca_ channels in a feedback loop by which cytosolic Ca^2+^ elevations regulate I_Ca_ channels (Figure 9A).

3. The present findings can also be explained by an alternative model in which *cpk3cpk6* mutation affects gene expression in such a manner that ABA activation of I_Ca_ channels is impaired. Similarly, the effects of the dominant *abi1–1* and *abi2–1* protein phosphatase 2C (PP2C) mutants on ABA responses could in principle result from indirect effects of these mutations on these ABA signal components, because these are dominant mutations and no pharmacological PP2C inhibitors are available to test effects of short-term PP2C impairment on channel regulation, as previously discussed [66]. This model in which *cpk3cpk6* may affect transcriptional or translational responses would be consistent with the activation of I_Ca_ currents in patch-clamped wild-type *Arabidopsis* guard cells without added cytoplasmic ATP and in the simultaneous presence of K252a (Figure 4J and 4K).

Experiments in V. faba guard cells show different responses than wild-type *Arabidopsis* guard cells that may shed light on the impairment in I_Ca_ channel activation in *cpk3cpk6* guard cells. In V. faba guard cells, K252a inhibits ABA activation of I_Ca_ currents and the protein phosphatase inhibitors calyculin A and okadaic acid activate I_Ca_ channel currents, suggesting that phosphorylation/dephosphorylation events regulate I_Ca_ channels in a parallel pathway in V. faba [96] (Figure 9). The present findings in *Arabidopsis* guard cells would predict a requirement for CDPK-dependent phosphorylation, prior to ABA activation of the ABA → NADPH oxidase → ROS → I_Ca_ channel regulation branch (Figures 4 and 9B). Previous analyses have indicated that *Arabidopsis* and *Vicia* guard cells emphasize different components of the guard cell signaling network, including differential phosphorylation-dependent responses [32,55,59,86,97,98]. In this respect, further comparative analyses among species should be useful in illuminating new signaling network mechanisms and branches.

Further studies will be necessary to distinguish the above models. The present study, however, provides a first characterization of a genetic mutation that impairs both Ca^2+^ activation of S-type anion channels and ABA activation of Ca^2+^-permeable I_Ca_ channels, indicating closer interactions among mechanisms that regulate these two classes of ion channels in guard cells than previously modeled.

### Conclusions

In summary, in this study, we provide direct molecular genetic evidence for Ca^2+^ sensors that function as positive transducers in stomatal ABA signaling and demonstrate functions of *CPK6* and *CPK3* in plant ion channel regulation in defined guard cell signal transduction elements. We functionally characterize disruption mutations in the two guard cell–expressed *CDPK* genes, *CPK3* and *CPK6*. These *CDPKs* function in ABA and Ca^2+^ activation of S-type anion channels and in ABA regulation of Ca^2+^-permeable I_Ca_ cation channels but not in R-type anion channel activity in guard cells. Furthermore, *cpk3cpk6* double mutants partially impair ABA-induced stomatal closure and Ca^2+^-reactive stomatal closure but not long-term Ca^2+^-programmed stomatal closure. Partial stomatal closing responses and differential regulation of R- and S-type anion current activities in *cpk3cpk6* mutant guard cells are consistent with models proposing parallel signaling mechanisms in a branched guard cell signal transduction network. Future cell-type–specific analyses of CDPKs may illuminate further cellular functions of this protein kinase family in plants.

## Materials and Methods

### Plant growth.

Arabidopsis thaliana plants (Columbia ecotype) were grown in soil (Sungro Special blend Professional Growing Mix; Seba Beach, Alberta, Canada) in a growth room under a 16-h-light/8-h-dark cycle at a photon fluence rate of 75 μmol m^−2^ s^−1^ and a temperature of 20 °C.

### Identification of CDPK genes expressed in guard cells.

Degenerate oligomer-based RT-PCR was used to identify guard cell–expressed CDPK genes from guard cell–enriched cDNA libraries [55]. Degenerate oligomers were designed from two highly conserved regions that were selected from aligned CDPK peptide sequences: HRDLKPENF and DG(K/R)I(D/N)(Y/F)(E/S)EF. The degenerate oligomers used to amplify guard cell–expressed CDPK genes are as follows: 5′-caymgigayytiaarccigaraaytt-3′ and 5′-aaytcitciwaityiatiykiccrtc-3′. Total RNA was extracted from guard cell–enriched epidermal strips as described [55] with the TriZOL reagent (Invitrogen, Carlsbad, California, United States). cDNA was synthesized using the First-Strand cDNA Synthesis Kit (Amersham-Pharmacia Biotech, Little Chalfont, United Kingdom). PCR was performed as described [55]. PCR products were cloned into the pGEM-T Easy vector (Promega, Madison, Wisconsin, United States). The sequences of the cloned PCR products were determined by regular sequencing reactions. Homozygous *cpk3* and *cpk6* single and double mutants were identified using gene-specific primers and primers that match T-DNA sequences as described previously [53,55].

### Cell-type–specific expression analyses and RT-PCR.

GCPs and mesophyll cell protoplasts were isolated by enzymatic digestion from 4-wk-old *Arabidopsis* plants as described in [54]. Total RNA was extracted from GCPs and mesophyll cell protoplasts with TriZOL reagent. Gene-specific primers for CPK3 and CPK6 were localized downstream of the DNA insertion sites as indicated (Figure 1B). For analyses of *KAT1* (AGI No.: At5g46240), *CBP* (AGI No.: At4g33050), and *ACTIN2* (AGI No.: At3g18780) mRNAs, gene-specific primers were used for amplifications.

### Patch-clamp analyses.

S- and R-type anion and I_Ca_ channel currents in *Arabidopsis* guard cells were recorded as published previously and described below (S-type: [9], R-type and I_Ca_: [65]). Patch-clamp data were recorded using an Axopatch 200 amplifier and pClamp software and analyzed with Axograph software (Axon Instruments, Union City, California, United States).

To measure whole-cell S-type anion currents, the pipette solution contained 150 mM CsCl, 2 mM MgCl_2_, 6.7 mM EGTA, 5 mM MgATP, 10 mM HEPES-Tris (pH 7.1), and CaCl_2_ to result in 2 μM free Ca^2+^. The bath solution contained 30 mM CsCl, 2 mM MgCl_2_, 1 mM CaCl_2_, and 10 mM MES-Tris (pH 5.6). Guard cells were extracellularly preincubated in the same solution with 40 mM CaCl_2_ added prior to patch-clamping (Figure 2), as previously described [9]; except for in ABA regulation analyses in which guard cells were preincubated in a solution containing 1 mM Ca^2+^ (Figure 3). Pretreatment of protoplasts with 40 mM CaCl_2_ allows analyses of Ca^2+^ activation of S-type anion channel currents in *Arabidopsis* (see [9] for details), and pretreatment with 1 mM extracellular Ca^2+^ allows analyses of ABA regulation of S-type anion channels while cytosolic Ca^2+^ is elevated, as previously shown [55]. Osmolarity of the solutions was adjusted with sorbitol to 500 and 485 mmol kg^−1^, respectively. The liquid junction potential was 0.5 mV. The membrane voltage was stepped from the holding potential of +35 mV to −145 mV for 40 s in −30-mV decrements [5]. The interpulse period was 12 s. No leak subtraction was performed. Recordings were made 7 to 10 min after access to the whole-cell configuration. For ABA activation of S-type channels, ABA was added to the bath solution prior to patch-clamping and seal formation. To test the robustness of ABA insensitivity in *cpk* mutant guard cells, 50 μM ABA was applied to guard cells.

To measure I_Ca_ channel currents, the pipette solution contained 10 mM BaCl_2_, 0.1 mM DTT, 4 mM EGTA, and 10 mM HEPES-Tris (pH 7.1), and the bath solution contained 100 mM BaCl_2_, 0.1 mM DTT, and 10 mM MES-Tris (pH 5.6). Guard cells were not preincubated in 40 mM CaCl_2_ in these experiments. Osmolarity of the solutions was adjusted with sorbitol to 500 and 485 mmol kg^−1^, respectively. NADPH (5 mM) was added to the pipette for the measurement of ABA-activated I_Ca_ currents. ABA was added extracellularly 16 min after whole-cell recordings began. In typical experiments, activation of I_Ca_ was observed within 1 min of ABA addition and continued for longer than 30 min. The voltage was ramped from −18 to −198 mV (after liquid junction potential correction) with a ramp speed of 180 mV s^−1^. In a standard measurement, the ramp protocol was applied 16 times to obtain an average current for a cell. The interpulse period was 1 min. When H_2_O_2_ (5 mM) activation of I_Ca_ was measured, the same procedures, analysis of average currents, and timing of application were used as for ABA regulation experiments.

To measure R-type anion channel currents, the pipette solution contained 75 mM K_2_SO_4_ [99], 2 mM MgCl_2_, 5 mM EGTA, 2.5 mM CaCl_2_, and 10 mM HEPES-Tris (pH 7.2) and the osmolarity was adjusted to 500 mmol kg^−1^. The bath solution contained 50 mM CaCl_2_, 2 mM MgCl_2_, and 10 mM MES-Tris (pH 5.6), and the osmolarity was adjusted to 485 mmol kg^−1^ with sorbitol. The voltage was ramped from the holding potential of 0 mV to −200 mV with a ramp speed of −20 mV s^−1^. Significance of differences between data sets was assessed by noncoupled double-tailed Student's *t*-test analysis.

### Stomatal movement analyses.

To measure changes in stomatal aperture, excised rosette leaves from 3- to 4.5-wk-old *Arabidopsis* plants were floated on 3 ml of opening buffer (5 mM KCl, 50 μM CaCl_2_, and 10 mM MES-Tris [pH 5.6]) for 2.5 h at 20 °C under light (photon fluency rate of 125 μmol m^−2^ s^−1^), so that the opening solution was taken up through petioles. After 2.5 h, ABA or CaCl_2_ was added to the opening buffer. For Ca^2+^-induced stomatal closing assays, opening solution without added CaCl_2_ was used. Leaves were incubated for an additional 3 h, and in the case of *cpk3-2cpk6–2,* for 3 h and for 45 min (Figure 6), after addition of Ca^2+^. Similar impairment in Ca^2+^-induced stomatal closure was found in *cpk3-2cpk6–2* double mutant plants compared to wild-type after both 45 min. and 3 h. Furthermore, similar stomatal responses were observed in *cpk3-2cpk6–2* plants 45 min. and 3 h after stimulation in parallel experiments (*p* > 0.50 at 0 μM CaCl_2_, *p* > 0.50 at 10 μM CaCl_2_, *p* > 0.45 at 100 μM CaCl_2_ (45 min versus 3 h *cpk3-2cpk6–2* data). Leaves were blended in opening solution in a Waring commercial blender (Waring Commercial, Torrington, Connecticut, United States) for 30 s. The blended material was filtered on a 100-μm nylon mesh, and epidermal strips were placed onto a microscope slide with a glass coverslip. The ratio of stomatal width to length was measured with an inverted microscope. Width was measured as the distance between the inner walls of the stomata, and length was measured as the length of the guard cells [100,101]. Data from stomatal ratios mirrored stomatal width data (Figure 6). For each sample, 20 to 30 stomata were measured. Blind experiments were also conducted, in which the genotype of leaves (wild-type or *cpk3 cpk6* mutant and the added stimuli) was unknown to the experimenter.

### Imposed Ca^2+^ oscillation responses.

To measure imposed Ca^2+^ oscillation–induced stomatal closure, epidermal peels were prepared by using Hollister medical adhesive (Stock No. 7730; Hollister Inc., Libertyville, Illinois, United States) to attach a leaf abaxial side down onto a coverslip. A razor blade was used to carefully remove the cuticle and mesophyll layers of the leaf, leaving the lower leaf epidermal layer containing stomatal complexes intact. The coverslip was then sealed with grease to the bottom of a custom microscope slide with a 2-cm-diameter hole cut in the center, creating a 200-μl well. The well was then filled with depolarizing buffer (50 mM KCl and 10 mM MES-Tris [pH 5.6]), and the leaf segment was incubated for 2.5 h in light (125 μmol m^−2^ s^−1^) to open stomata. [Ca^2+^]_cyt_ transients were imposed by rapid exchange of the chamber buffer between depolarizing buffer and hyperpolarizing buffer (1 mM KCl, 1 mM CaCl_2_, 10 mM MES-Tris [pH 5.6]). Depolarizing buffer decreases [Ca^2+^]_cyt_ levels, whereas hyperpolarizing buffer increases [Ca^2+^]_cyt_ levels [68]. For each experiment, four separate transients with an approximately 10-min period were imposed. Stomatal apertures of several individually mapped stomata were measured at the indicated time points for 3 h after the start of imposed oscillations.

Cytosolic Ca^2+^ concentration changes during imposed [Ca^2+^]_cyt_ transient experiments were confirmed by using the improved Yellow Cameleon 3.6 (YC3.6) fluorescence reporter [102]. Col-0 and *cpk3-1cpk6–1* plants were transformed with YC3.6. Based on calibration measurements, the imposed Ca^2+^ transients using YC3.6 corresponded to average cytoplasmic Ca^2+^ baseline levels of approximately 0.15 μM and average peak levels of approximately 0.6 μM. The imposed oscillation protocol that was applied subjects cells to large changes in both the external Ca^2+^ and Cl^−^ concentrations. Earlier versions of cameleon were sensitive to the Cl^−^ concentration, as Cl^−^ affects the pH sensitivity of the yellow fluorescent protein chromophore [103] and therefore previous studies were performed with yellow cameleon YC2.1 which has important amino acid substitutions (V68L and Q69K) that substantially reduce sensitivity to the interacting Cl^−^ and H^+^ ions [104,105]. In the present study, YC3.6 was used, which also has important amino acid substitutions that substantially reduce sensitivity to the interacting Cl^−^ and H^+^ ions and a greatly enhanced ratio changes in response to physiological [Ca^2+^]_cyt_ changes [102]. Previous work with YC2.1 showed that the ratio changes observed during imposed oscillations are due to the changes in calcium, not changes in Cl^−^, as imposing the same chloride changes while removing extracellular Ca^2+^ does not produce any YC2.1 ratio changes (supplemental data to [29]; http://www.nature.com/nature/journal/v411/n6841/extref/4111053aa.html). In these experiments, extracellular Ca^2+^ was chelated with 10 mM EGTA, which lowers external Ca^2+^ to nanomolar levels, to physically inhibit Ca^2+^ influx that would otherwise occur at strongly hyperpolarized potentials. In further controls, replacing CaCl_2_ with MgCl_2_ in the bath did not produce cameleon ratio changes [106]. Further control experiments were performed with the new reporter YC3.6 in which Ca^2+^ changes were imposed but not Cl^−^ changes, by using the impermeant counteranion imminodiacetate (Figure 10). Stomata were extracellularly perfused alternately with solutions containing either 100 mM K^+^ imminodiacetate (no added Ca^2+^) (pH 6.15) or 1 mM K^+^ imminodiacetate and 1 mM Ca^2+^ imminodiacetate (pH 6.15). These controls showed YC3.6 ratio changes in the absence of imposed Cl^−^ changes, demonstrating that Ca^2+^ changes are being reported (Figure 10), as previously shown for YC2.1 [29,104,105].

**Figure 10:**
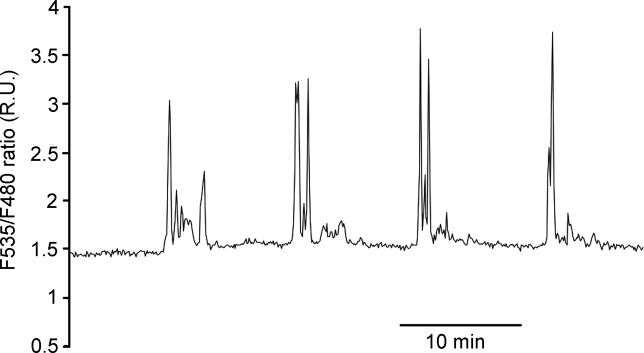
Alterations in the Extracellular Calcium Concentration in the Absence of Changes in the Extracellular Cl^−^ Concentration Cause Large Ratio Changes in YC3.6 in *Arabidopsis* Guard Cells Illustrating that YC3.6, Like YC2.1, Reports Cytosolic Ca^2+^ Changes and Not Cl^−^ Changes during Imposed Transients See Materials and Methods. The YFP/CFP emission ratio is monitored in a guard cell (wild-type) expressing YC3.6. Ratio increases accompany exposure to high extracellular calcium using the membrane-impermeable counteranion, imminodiacetate.

### Germination assays.

Germination assays were performed as described previously [107]. Mutant and wild-type seeds were harvested at the same time and then sterilized using chlorine. Sterilized seeds were plated on 1/2 MS medium containing MES (0.5 g/L) (pH 5.7), supplemented with 0, 0.3, 1, and 5 μM ABA. Plates were kept in the cold room for 2 d and then transferred to a growth room. Seed germination was scored on days 3, 5, 7, and 11.

## Abbreviations

AGI No.: *Arabidopsis* gene identifier number
[Ca^2+^ ]_cyt_: cytosolic free Ca^2+^ concentration
ABA: abscisic acid
CDPK: calcium-dependent protein kinase
GCP: guard cell protoplast
I_Ca_: Ca^2+^- permeable channel current
K252a: (8*R**,9*S**,11*S**)-(−)-9-hydroxy-9-methoxycarbonyl-8-methyl-2,3,9,10-tetrahydro-8,11,epoxy-1*H*,8*H*,11*H*-2,7*b*,11*a*-triazadibenzo[*a,g*]cyclo-octa[*c,d,e*]-trinden-1-one
PP2C: protein phosphatase 2C
R-type: rapid-type anion channel
ROS: reactive oxygen species
RU: ratio units
S-type: slow-type anion channel
YC3.6: yellow Cameleon 3.6
